# MRE11A: a novel negative regulator of human DNA mismatch repair

**DOI:** 10.1186/s11658-024-00547-z

**Published:** 2024-03-14

**Authors:** Demin Du, Yueyan Yang, Yuanyuan Zhang, Guanxiong Wang, Liying Chen, Xiaowei Guan, Lene Juel Rasmussen, Dekang Liu

**Affiliations:** 1 Department of Human Anatomy and Histoembryology, Nanjing University of Chinese Medicine, Nanjing, 210023 China; 2 Affiliated Hospital of Nanjing University of Chinese Medicine, Nanjing, 210023 China; 3 Center for Healthy Aging, Department of Cellular and Molecular Medicine, University of Copenhagen, 2200 Copenhagen, Denmark

**Keywords:** Alkylating agents, DNA mismatch repair, DNA repair, MRE11A, PMS2

## Abstract

**Background:**

DNA mismatch repair (MMR) is a highly conserved pathway that corrects DNA replication errors, the loss of which is attributed to the development of various types of cancers. Although well characterized, MMR factors remain to be identified. As a 3′–5′ exonuclease and endonuclease, meiotic recombination 11 homolog A (MRE11A) is implicated in multiple DNA repair pathways. However, the role of MRE11A in MMR is unclear.

**Methods:**

Initially, short-term and long-term survival assays were used to measure the cells’ sensitivity to *N*-methyl-*N*′-nitro-*N*-nitrosoguanidine (MNNG). Meanwhile, the level of apoptosis was also determined by flow cytometry after MNNG treatment. Western blotting and immunofluorescence assays were used to evaluate the DNA damage within one cell cycle after MNNG treatment. Next, a GFP-heteroduplex repair assay and microsatellite stability test were used to measure the MMR activities in cells. To investigate the mechanisms, western blotting, the GFP-heteroduplex repair assay, and chromatin immunoprecipitation were used.

**Results:**

We show that knockdown of MRE11A increased the sensitivity of HeLa cells to MNNG treatment, as well as the MNNG-induced DNA damage and apoptosis, implying a potential role of MRE11 in MMR. Moreover, we found that MRE11A was largely recruited to chromatin and negatively regulated the DNA damage signals within the first cell cycle after MNNG treatment. We also showed that knockdown of MRE11A increased, while overexpressing MRE11A decreased, MMR activity in HeLa cells, suggesting that MRE11A negatively regulates MMR activity. Furthermore, we show that recruitment of MRE11A to chromatin requires MLH1 and that MRE11A competes with PMS2 for binding to MLH1. This decreases PMS2 levels in whole cells and on chromatin, and consequently comprises MMR activity.

**Conclusions:**

Our findings reveal that MRE11A is a negative regulator of human MMR.

**Graphical Abstract:**

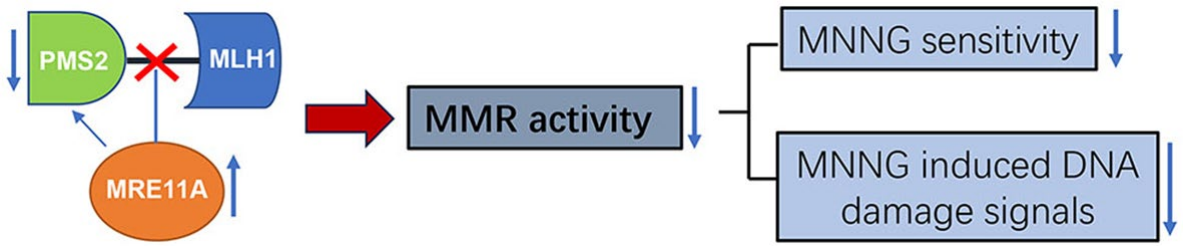

**Supplementary Information:**

The online version contains supplementary material available at 10.1186/s11658-024-00547-z.

## Background

High fidelity of DNA replication is critical for maintaining genomic integrity during cell proliferation. DNA mismatch repair (MMR) is an important DNA repair pathway that plays a critical role in DNA replication fidelity. MMR encompasses three key steps: mismatch recognition, mismatch removal, and DNA strand resynthesis. In human cells, the recognition and binding of mismatches are mediated by two distinct heterodimers: MutSα (MSH2-MSH6) and MutSβ (MSH2-MSH3). MutSα primarily identifies and interacts with base–base mismatches, while MutSβ targets larger insertion/deletion loops (IDLs). Upon recognition, MutLα (MLH1-PMS2) is recruited by MutSα/β complexes, leading to the formation of a tetrameric sliding clamp complex. This complex generates nicks in close proximity to the mismatches. Subsequently, exonuclease 1 (EXO1) acts to excise the strand harboring the mismatch, resulting in a gap. This gap is then filled by DNA polymerase δ, while nicks are religated by DNA ligase I [1, 2]. Loss of MMR led to hypermutational phenotypes in human cells that contributed significantly to the early onset of various types of cancers [3, 4]. In the clinic, MMR status is widely used as a biomarker for diagnosis and medication choice for cancer treatment. For instance, MMR-deficient tumors exhibit resistance to chemotherapeutic drugs such as alkylating agents [5–9]. In addition, MMR-deficient tumors are sensitive to immunotherapy, i.e., anti-PD-1 or PD-L1 treatment [10–12]. As such, evaluating MMR activity in tumors is critical for timely diagnosis and effective treatment of cancer. For assessing MMR status, immunostaining of established MMR factors and microsatellite instability (MSI) testing are mostly used in clinics [13]. However, alterations in known MMR proteins cannot be detected in a large proportion of MSI cancers [5, 14], indicating the existence of unknown MMR factors. Considering that EXO1 deficiency only causes milder MMR-deficient phenotypes compared with the inactivation of MSH2 or MLH1 [15–17], we initiated a screening to identify novel exo/endonucleases in MMR other than EXO1 [18]. We found that knockdown of meiotic recombination 11 homolog A (MRE11A) enhanced the sensitivity of cells to the alkylating drug *N*-methyl-*N*′-nitro-*N*-nitrosoguanidine (MNNG).

The MRE11A protein possesses 3′–5′ nuclease and endonuclease activities. Within cells, MRE11A forms the MRN complex by associating with two partner proteins, RAD50 and NBS1. This complex serves as an early sensor and processor of DNA double-strand breaks (DSBs) and plays crucial roles in various chromosome-related processes, including DNA homologous recombination, telomere metabolism, DNA replication fork processing, and meiosis. Additionally, MRE11A is involved in break-induced repair/replication (BIR) and interstrand crosslink (ICL) repair [19–21]. Moreover, its interaction with MLH1 suggests its potential involvement in mismatch repair (MMR) [22, 23]. Previous reports have described reduced MMR activity on 3′ nicked DNA substrates in vitro in MRE11A-deficient cells [24]. However, other studies have argued that MMR deficiency may also impact MRE11A expression or functions [25–29]. Conversely, a subsequent investigation employing murine cells demonstrated that inactivation of MRE11A did not affect MMR activity [30]. These divergent findings prompted us to conduct further investigations to characterize the precise role of MRE11A in MMR.

Here, we show that inactivation of MRE11A increases cell sensitivity to MNNG and demonstrate that MRE11A negatively regulates MMR activity by competing with PMS2 for binding to MLH1.

## Methods

### Cell line generation

HeLa cells were purchased from CELLCOOK (cat. no. CC1101), and STRs were identified in advance. All cells were grown in Dulbecco’s modified Eagle’s medium (DMEM; Bioind) containing 10% fetal bovine serum (Bioind) and 1% penicillin/streptomycin (Gibco) at 37 °C in 5% CO_2_. MRE11 knockdown cell lines were generated by lentivirus obtained from GeneChem. Two different target sequences were used (siMRE11-1: 5′-GTACGTCGTTTCAGAGAAA-3′, siMRE11-2: 5′-GGAGGATATTGTTCTAGCT-3′).

### Gene overexpression and knockdown

MRE11A (NM_001330347) and PMS2 (NM_000535) overexpression constructs were obtained through gene synthesis and cloned and inserted into pcDNA3.1(+) by Gene Universal.

Flag-MRE11A and Flag-MRE11A (452–634 aa) cassettes were achieved by PCR using the following primers: Fwd 5′-GGGGTACCgccaccatgGATTACAAGGACGACGATGACAAGagtactgcagatgcact-3′ and Rev 5′-AATGCGGCCGC TTATCTTCTATTTCTTCTTAAAG-3′ for Flag-MRE11 and Fwd 5′-GGGGTACCgccaccatgGATTACAAGGACGACGATGACAAGagagggatgggtgaagcagt 3′-and Rev 5′-AATGCGGCCGCTTAATTTCGGGAAGGCTGCTGTC-3′ for Flag-MRE11 (452–634 aa). The PCR products were then subcloned and inserted into the pcDNA3.1(+) plasmid. For each six-well plate, 2 μg plasmid was transfected using PolyJet™ in vitro DNA transfection reagent (SignaGen, SL100688) according to the manufacturer’s instructions.

All siRNAs were synthesized by RiboBio according to the following target sequences: siMRE11A-1: 5′-GTACGTCGTTTCAGAGAAA-3′, siMRE11-2: 5′-GGAGGATATTGTTCTAGCT-3′, siMSH2-1: 5′-GCTAAAAGCTGAAGTAATA-3′, siMSH2-2: 5′-GGAGGTAAATCAACATATA-3′, siMLH1-1: 5′-CTGAGATGCTTGCAGACTA-3′, siMLH1-2: 5′-GGAAGATGGTCCCAAAGAA-3′, siNC (negative control): 5′-TTCTCCGAACGTGTCACGT-3′. siRNA transfection followed the general guidelines of reverse transfection using Lipofectamine^®^ 2000 (Thermo Scientific, 11,668,019). In brief, 5 μl Lipofectamine^®^ 2000 and 100 pm siRNA were diluted in 200 μL Opti-MEM™ I reduced serum medium (Thermo Scientific, 31,985,062) for 5 min at RT, diluted Lipofectamine^®^ 2000 and siRNA were added to the six-well plate, mixed gently, and incubated for 15 min at room temperature. After that, 2 ml complete growth medium without antibiotics with 1 × 10^6^ cells was added to the plate and mixed gently. The cells were incubated at 37 °C with 5% CO_2_ and harvested after 96 h.

### RNA extraction and RT-qPCR analyses

A total RNA isolation kit was purchased from Vazyme (RC101). For RT-qPCR, RNA was reverse transcribed to cDNA by reverse transcriptase (Vazyme, R312-01). qPCR analyses were performed with Universal SYBR qPCR Master Mix (Vazyme, Q711-02). For the results analysis, GAPDH was used as a reference. The primers are listed below: MRE11A: Fwd 5′-ATCGGCCTGTCCAGTTTGAAA-3′ and Rev 5′-TGCCATCTTGATAGTTCACCCAT-3′. PMS2: Fwd 5′-TTTGCCGACCTAACTCAGGTT-3′ and Rev 5′-CGATGCGTGGCAGGTAGAA-3′. GAPDH: Fwd 5′-GGAGCGAGATCCCTCCAAAAT-3′ and Rev 5′-GGCTGTTGTCATACTTCTCATGG-3′.

### Western blotting and antibodies

Cell lysis solution was purchased from Beyotime (P0013B), containing 50 mM Tris(pH 7.4), 150 mM NaCl, 1% Triton X-100, 1% sodium deoxycholate, 0.1% SDS. Phosphatase inhibitor (Beyotime, P1081) and protease inhibitor (CWBIO, CW2200) were added just before use. For protein extraction, media was aspirated from plates, cells were washed thrice with cold phosphate-buffered saline (PBS), and lysis solution was added to the plate and placed on ice for 30 min. The lysis solution was immediately collected in a microcentrifuge tube and centrifuged at 12,000 rpm for 5 min (4 °C), and supernatants were collected as the total cell extracts. Protein levels were determined using a BCA protein assay kit (Beyotime, P0012). For western blotting, after sodium dodecyl sulfate polyacrylamide gel electrophoresis (SDS-PAGE) running, proteins were transferred to polyvinylidene fluoride (PVDF) membranes and blocked with TBST (137 mM NaCl, 20 mM Tris, 0.1% Tween 20) with 5% (w/v) skim milk for 1 h at room temperature. Next, the membrane was incubated with primary antibody in antibody dilution buffer (TBST containing 5% skim milk) overnight at 4 °C. The next day, the membrane was washed three times for 5 min each with TBST and incubated with the secondary antibody for 1 h at room temperature. Protein signals were visualized with ECL western blotting reagents (Vazyme, E412-02). The primary antibodies are as follows: Flag (Proteintech, 20543-1-AP), PMS2 (Proteintech, 66075-1-Ig), MSH6 (Proteintech, 18120-1-AP), EXO1 (Proteintech, 16253-1-AP), Histone-H3 (Proteintech, 17168-1-AP), CHK2 (Proteintech, 13954-1-AP), CHK1 (Proteintech, 25887-1-AP), MSH2 (Proteintech, 15520-1-AP), MRE11A (Proteintech, 10744-1-AP), actin (Proteintech, 20536-1-AP), P-CHK2-Thr68 (CST, #2661), P-CHK1- Ser345 (CST, #2348), 53BP1 (CST, #4937) and MLH1 (Affinity, DF6057). Secondary antibodies: HRP-conjugated Affinipure goat anti-rabbit IgG (SA00001-2) and HRP-conjugated Affinipure goat anti-mouse IgG (SA00001-1) were purchased from Proteintech.

### Chromatin extraction and coimmunoprecipitation

Chromatin extraction was performed with the Chromatin Extraction Kit (Abcam, #ab117152) according to the manufacturer’s instructions. Briefly, cells were trypsinized and washed twice with 10 mL cold PBS, and the cells were counted with a hemocytometer. Then, the cell pellet was harvested by centrifugation. Next, lysis buffer (200 µL/1 × 10^6^ cells) containing protease inhibitor was added to the cell pellet, and the cells were resuspended gently, incubated on ice for 10 min, and vortexed vigorously for 10 s. The supernatant was removed by centrifugation. The sediment was resuspended using extraction buffer (50 µL/1 × 10^6^ cells) containing protease inhibitor and incubated on ice for 10 min and vortexed occasionally, followed by sonication and centrifugation at 12,000 rpm at 4 °C for 10 min. The supernatants were collected as chromatin extracts. The proteins bound to chromatin were analyzed by western blotting.

For immunoprecipitation, extracted chromatin proteins were incubated with MSH2 antibody (Proteintech, 15520-1-AP) or normal rabbit IgG (CST, #2729) overnight at 4 °C and then with Protein G magnetic beads (MCE, HY-K0204) for 1 h at RT followed by washing three times with PBST (1 × PBS with 0.5% Tween-20, pH 7.4). Then, the beads were separated using magnetic separation rack, and the beads were washed three times with PBST again. Then, 1 × SDS‒PAGE loading buffer was added, and the sample was heated to 98 °C for 5 min. Finally, the beads were separated by centrifugation, and the supernatant containing proteins was transferred to a new vial. The products were analyzed by western blotting.

### Immunofluorescence

The cells were plated on six-well cell culture plates with coverslips. To assess 53BP1 foci, cells were treated with O6-benzylguanine (O6-BG) (Sigma) for 1 h and then treated with MNNG for 12 h. Following MNNG treatment, cells were fixed with 4% paraformaldehyde for 10 min, permeabilized in PBS buffer containing 0.1% Triton X-100 for 15 min. After that, cells were incubated with 53BP1 antibody (Cell Signaling, #4937) and CYCLIN A antibody (Santa, sc-271645) overnight at 4 °C and then stained with Alexa Fluor 488 or Alexa Fluor 555 secondary antibodies (Invitrogen) for 1 h at RT. Nuclei were counterstained with 4′,6-diamidino-2-phenylindole (DAPI). Finally, coverslips were mounted with anti-fading (Solarbio). Fluorescence images were taken by a Leica DM6 B fluorescence microscope.

### Cell survival, growth, and apoptosis analysis

MNNG and O6-BG were dissolved in dimethyl sulfoxide (DMSO) and stored at −20 °C. HeLa cells were treated with O6-BG (10 μM) for 1 h prior to the addition of MNNG at the indicated concentration.

To assess survival, cells were washed and harvested after treatment with the indicated concentrations of MNNG for 72 h and resuspended in 1 ml of PBS. Then, the optical density (OD) was measured at 600 nm using an iMark™ microplate absorbance reader (Bio-Rad).

For the clonogenic assay, HeLa cells were transfected with scrambled or targeted siRNA, and cells were plated on six-well plates 1 day before treatment with the indicated concentrations of MNNG. Approximately 8 days after treatment, the cells were stained with 0.5% Crystal Violet in 20% ethanol. Only colonies containing > 100 cells were counted.

To measure the growth rate, suspended cells were counted using a hemocytometer, diluted to 10,000 cells per milliliter, and then inoculated into a 96-well plate (100 μL per well) in sextuplicate. After 24, 48, 72, and 96 h of incubation at 37 °C, 10 μL of CCK-8 (Vazyme, A311-01) was added to each well for a 1 h incubation. After that, the absorbance (OD) of the plate was measured at 450 nm by the iMark™ microplate absorbance reader (Bio-Rad). The growth rate was defined as the OD ratio to the OD at 24 h.

To detect apoptosis, cells were treated with MNNG, harvested after 72 h, washed twice, and suspended in binding buffer. Then, the cells were stained with an Annexin V-FITC/PI apoptosis detection kit (Vazyme, A211-01), and the cell distribution was detected using a Beckman Coulter Gallios (Beckman Coulter). The results were analyzed by using Kaluza Analysis 2.1 (Beckman Coulter).

### Microsatellite analysis and MMR assay

We isolated single cell using the limiting dilution approach and grew it for approximately 30 generations to permit mutation accumulation. We then collected ten clones from each treated group and sent them to Shanghai Personalbio Technology Co., Ltd. for MSI analysis based on the fluorescent PCR amplification of microsatellite genes, including NR-21, NR-24, BAT-25, BAT-26, and MONO-27, and capillary electrophoresis analysis by a 3730xl DNA analyzer. The results were demonstrated by GeneMaker software.

For the MMR assay in cells, substrate heteroduplex GFP substrate was prepared according to Zhou et al. [31]. Briefly, p111 was nicked with Nb. Bpu10I (Thermo Scientific) and further digested with ExoIII (NEB) to generate single-strand circular DNA. p189 was linearized by SphI (NEB). To obtain the GFP-heteroduplex, linearized double-strand DNA was annealed with p111 single-strand circular DNA in 1X annealing buffer (Beyotime, #D0251) by heating at 95 °C for 5 min, followed by slow cooling from 95 to 25 °C within 45 min. The annealing product was then treated with Plasmid-Safe ATP-Dependent DNase (Biosearch Technologies). To assess MMR activity, HeLa cells were transfected with 1 μg of the heteroduplex plasmid and 0.8 μg of pmCherry-C1. After incubation for 24 h, the cells were harvested and analyzed for fluorescence intensity with a Beckman Coulter Gallios (Beckman Coulter). The result was analyzed by using Kaluza Analysis 2.1 (Beckman Coulter), and the relative repair efficiency was measured by the ratio of GFP-positive cells to mCherry-positive cells.

### Statistical analyses

All data are presented as mean ± standard deviation (SD) and were analyzed by unpaired *t* tests. Light and fluorescence microscopy photos were captured by Leica LAS X software. ImageJ was used for intensity quantifications of blotting assays, and the fluorescence-activated cell sorting (FACS) data were analyzed and illustrated by using Kaluza Analysis software. Statistical analyses were performed by GraphPad Prism 8. Statistical significance was set when *P* < 0.05 (two sided).

## Results

### MRE11A knockdown increases the sensitivity of HeLa cells to MNNG

**Fig. 1:**
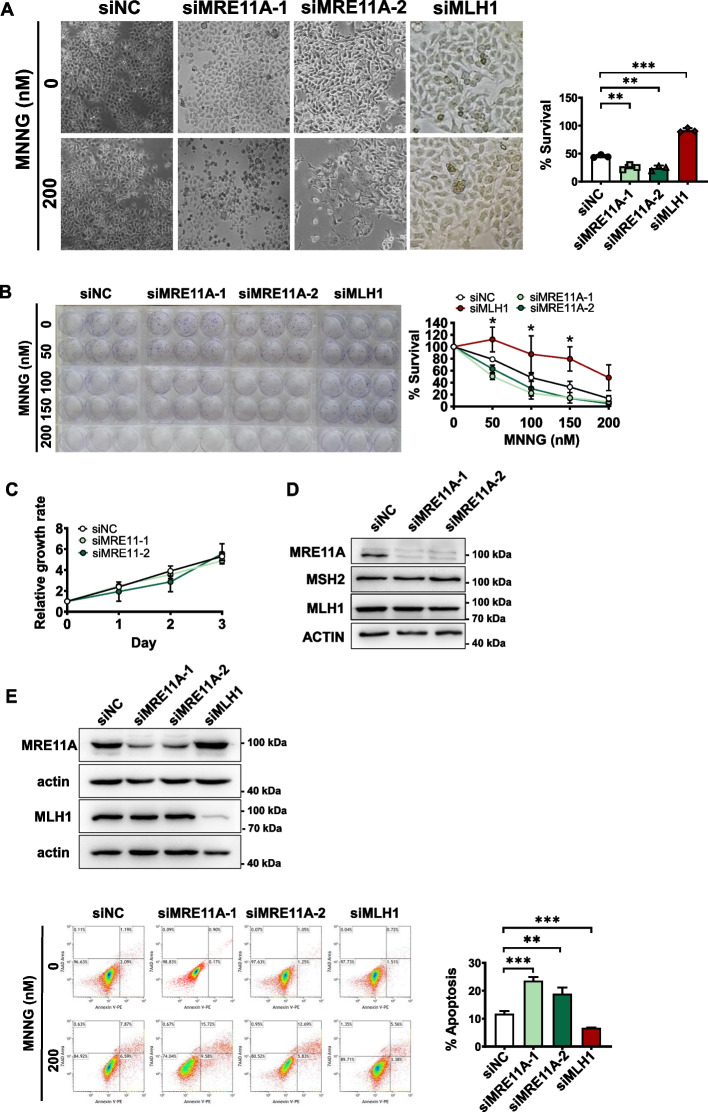
MRE11A deficiency sensitizes cells to MNNG treatment. **A** HeLa cells were transfected with two different siRNAs targeting MRE11A and exhibited abnormal morphology and reduced survival 72 h after 200 nM MNNG treatment. The survival rate was the percentage of surviving cells to parallel cells treated only with O6-benzylguaine and DMSO in each group, and cells with MLH1 deficiency were used as a positive control. **B** MRE11A knockdown cells were treated with MNNG and seeded in triplicate in six-well plates, and after approximately 2 weeks, the cells were stained with Crystal Violet, and the colonies with ≥ 200 cells were counted. The survival rate was the percentage of surviving clones in parallel wells treated only with DMSO in each group, and cells with MLH1 deficiency were used as a positive control. **C** The growth rates of control cells and MRE11A knockdown cells were measured in 96-well plates with CCK8 reagents. **D** Western blotting showed no significant changes in the protein levels of MSH2 and MLH1 in MRE11A knockdown cells. **E** Representative flow cytometry pictures of scatter plots of PI versus Annexin V staining of the siNC control, MRE11A and MLH1 knockdown cells 72 h after 200 nM MNNG treatment. The right graph shows the statistical analysis of the left flow cytometry data, quantification, and comparison of the proportions of apoptotic cells in each group. The % apoptosis was calculated as the % apoptosis of cells with 200 nM MNNG minus that with only DMOS treatment. All data were analyzed with an unpaired two-tailed Student’s *t* test. Data are shown as mean ± SD, *n* = 3, *P < 0.05, **P < 0.01, ***P < 0.001

In the absence of MGMT, MNNG can induce the formation of O(6)-methylguanine (^O(6)Me^G) lesions. During genomic replication, these ^O(6)Me^G:T mispairs are recognized by MutSα. If^O(6)Me^Gs are present on the template (mother) strand, they can lead to a phenomenon known as the “futile cycle” in the mismatch repair (MMR) process. In this cycle, thymine is repeatedly excised and misincorporated opposite the O(6)MeG lesion. Consequently, nicks or gaps may persist, which can ultimately result in the collapse of the replication fork during the second round of genomic replication. This, in turn, activates a G2 checkpoint and subsequently leads to cell cycle arrest [18, 32, 33]. Hence, higher MMR activity correlates with increased sensitivity to MNNG in cells. In our screening assay, we observed that cells depleted of MRE11A exhibited increased sensitivity to MNNG [18]. To confirm the significance of MRE11A as a relevant factor, we employed two distinct siRNA sequences targeting MRE11A in HeLa cells, siMRE11A-1 and siMRE11A-2. As controls, siNC (nontargeting siRNA) and siMLH1 were utilized in both assays. The viability of MRE11A-depleted cells was assessed 72 h post-MNNG treatment (O(6)-benzylguanine was added 1 h before treatment to inactivate MGMT), which corresponded to approximately three cell cycles. Our results, depicted in Fig. 1A, demonstrated that MRE11A-deficient cells exhibited abnormal morphology and lower survival rates compared with the siNC control following MNNG treatment, even at a concentration as low as 200 nM (*n* = 3, 3; *p* = 0.002, 0.002). In parallel, a clonogenic assay revealed that MRE11A knockdown significantly reduced the percentage of surviving cell colonies compared with the siNC control after MNNG treatment at concentrations of 50 nM (*n* = 3, 3; *P* = 0.0012, 0.016), 100 nM (*n* = 3, 3; *P* = 0.019, 0.087), and 150 nM (*n* = 3, 3; *P* = 0.071, 0.031) (Fig. 1B). Notably, there were no significant differences in the growth rate and protein expression levels of MSH2 and MLH1 after MNNG treatment between MRE11A knockdown and siNC control cells (Fig. 1C, D).

Upon exposure to MNNG, cells undergo two cell cycles before the mismatch repair (MMR) machinery can activate cell apoptosis pathways [33]. In our study, we observed that knockdown of MRE11A significantly increased apoptosis compared with the siNC controls (*n* = 3, 3; *P* < 0.001, =0.0069), while knockdown of MLH1, as expected, decreased apoptosis (*n* = 3; *P* < 0.001) (Fig. 1E). These findings collectively indicate that depletion of MRE11A heightens cellular sensitivity to alkylation damage, implying a potential regulatory role of MRE11A in MMR.

### MRE11A depletion enhances MNNG-induced DNA damage signals within one round of the cell cycle

**Fig. 2:**
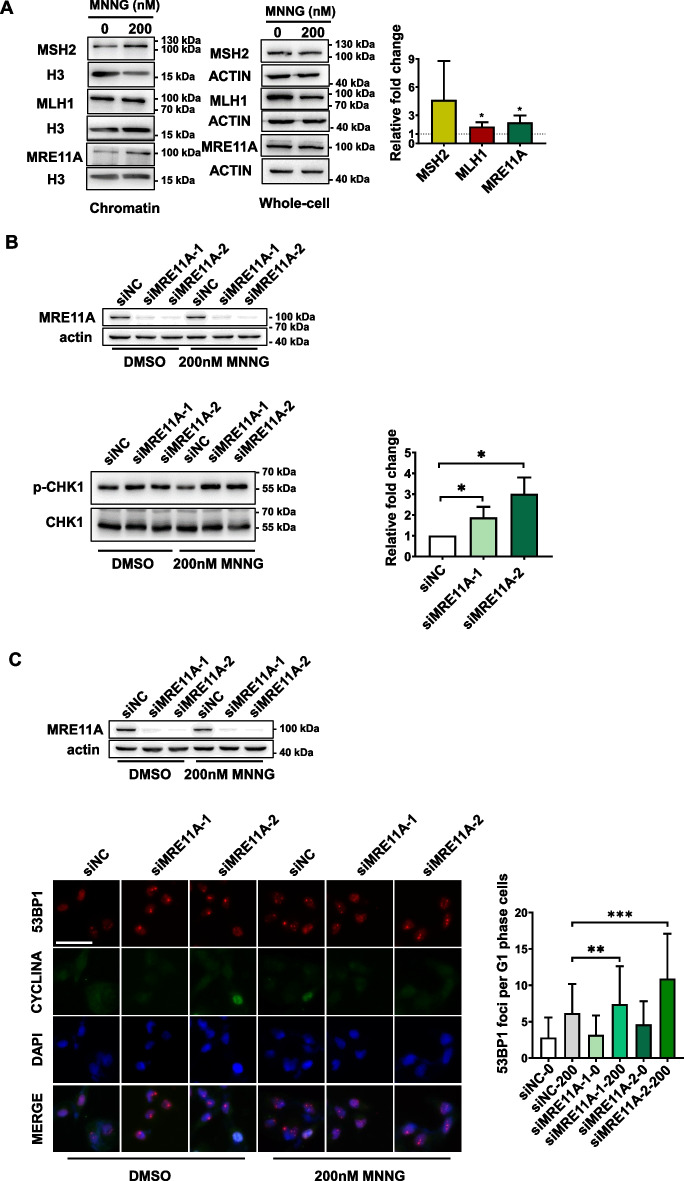
MRE11A deficiency increases DNA damage signals 12 h after MNNG treatment. **A** Representative western blotting pictures of chromatin binding and whole-cell MSH2, MLH1, and MRE11 proteins 12 h after DMSO or 200 nM MNNG treatment of HeLa cells. The level of histone H3 was set as an internal control. The right graph shows the quantification of the fold change in the ratio of chromatin binding to the whole-cell proteins MSH2, MLH1, and MRE11A after exposure to 200 nM MNNG. **B** Representative western blotting of the phosphorylation levels of CHK1 12 h after DMSO or 200 nM MNNG treatment. The alteration of phosphorylation level was calculated as p-CHK1 level normalized by total CHK1 protein after 200 nM MNNG treatment minus that with only DMSO treatment. The right graph shows the quantification of protein level changes relative to siNC. **C** Representative immunofluorescence images of 53BP1 foci in G1 phase 12 h after DMSO or 200 nM MNNG treatment. The right graph shows the quantification of the number of 53BP1 foci per cell in G1 phase (CYCLINA-). At least 250 cells were counted for each group. Data are shown as mean ± SD, *P < 0.05, **P < 0.01, ***P < 0.001, using unpaired two-tailed Student’s *t* test

MNNG-induced cell death is dependent on the activity of MMR to induce replication fork collapse in subsequent cell cycles [32, 33]. Additionally, MRE11A has been implicated in the repair of replication fork collapse [20, 21]. To investigate the specific role of MRE11A in MMR, while avoiding the interference of its role in replication fork collapse repair, we subjected HeLa cells to 12-h treatment with MNNG, within a single cell cycle (as shown in Fig. 1C, the doubling time of siNC, siMRE11A-1, and siMRE11A-2 was approximately 24 h). Notably, as shown in Fig. 2A, the recruitment of MRE11A to chromatin was already observed after 12 h of MNNG treatment (*n* = 3; *P* = 0.047), suggesting its involvement in processing alkylation damage with MMR machinery. To assess the extent of DNA damage resulting from MMR processing of alkylation damage in the first cell cycle following MNNG treatment, we measured the phosphorylation level of CHK1 and the number of 53BP1 foci in G1-phase cells [32, 34–37]. Our results demonstrated that MRE11A deficiency led to increased CHK1 levels and an elevated number of 53BP1 foci after MNNG treatment compared with the control (*n* = 3, 3; *P* = 0.041, 0.011, and 0.0040, < 0.001) (Fig. 2B, C). Furthermore, to consolidate our findings, we overexpressed MRE11A in HeLa cells, which resulted in reduced CHK1 phosphorylation levels (*n* = 3; *P* = 0.0015) and decreased numbers of 53BP1 foci (*n* = 3;* P* = 0.0053) compared with the controls (Additional file 1: Fig. S1). These results collectively suggest that MRE11A may prevent MNNG-induced DNA damage, potentially by interfering with MMR activity.

### MRE11A negatively regulates MMR activity

**Fig. 3:**
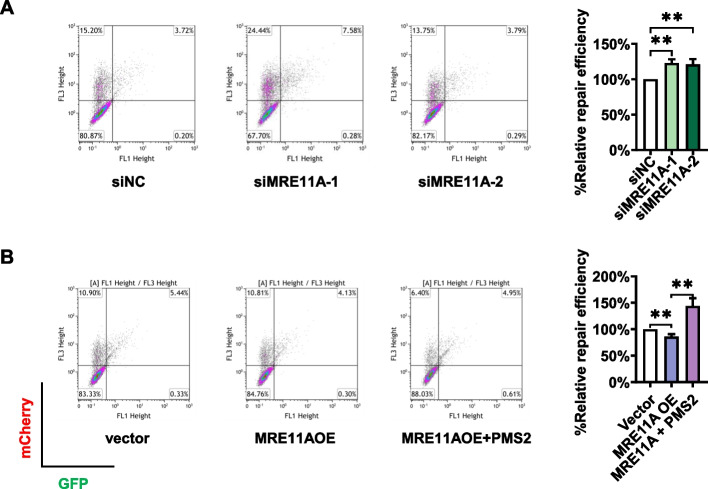
MRE11A negatively regulates MMR activity in HeLa cells. The left pictures represent the scatter plots of cells cotransfected with GFP-heteroduplex and mCherry plasmids as described in “Materials and Methods” section. The *x*-axis and *y*-axis represent the signal intensities of GFP and mCherry, respectively. The MMR repair efficiency was calculated as the ratio of the number of GFP-positive cells to mCherry-positive cells, and the quantification results relative to siNC or empty vector controls are shown in the right graphs. **A** Cells were transfected with siNC or two MRE11A siRNAs followed by GFP-heteroduplex and mCherry plasmid cotransfection after 2 days. The next day, the cells were subjected to flow cytometry for GFP and mCherry signal analysis. **B** Cells were transfected with empty vector, MRE11A overexpression plasmid (MRE11AOE), or MRE11A + PMS2 overexpression plasmids (MRE11OE + PMS2) followed by GFP-heteroduplex and m-cherry plasmid cotransfection after 2 days. The next day, the cells were subjected to flow cytometry for GFP and mCherry signal analysis. Data are shown as mean ± SD, *P < 0.05, **P < 0.01, ***P < 0.001, using unpaired two-tailed Student’s *t* test

To directly detect the influence of MRE11A on MMR activity, we employed a GFP-heteroduplex assay, which utilizes the expression of GFP as an indicator of successful heteroduplex repair. To normalize transfection efficiency across all experiments, mCherry plasmids were cotransfected [31]. Our findings revealed that knockdown of MRE11A resulted in an increase in MMR activity (*n* = 3, 3; *P* = 0.0015, 0.0087, Fig. 3A), while its overexpression led to a decrease in MMR activity (*n* = 3, 3; *P* = 0.0067, Fig. 3B). These results are consistent with our above observations that MRE11A served as a negative regulator of MMR-induced DNA damage signals and influenced cell survival following MNNG treatment (Figs. 1, 2).

Microsatellite instability (MSI) serves as an indirect indicator of MMR deficiency [38]. We established stable cell lines of MRE11A knockdown or overexpression, duplicated single cell approximately 30 times (approximately 30 days), and harvested genomic DNA to assess the insertion/deletion mutations of microsatellite markers, BAT25, BAT26, MONO27, NR21, and NR24. Surprisingly, no changes were observed in these microsatellite markers (Additional file 1: Fig. S2A), while MSI was detected in the positive control of MLH1-deficient HEK293T cells (Additional file 1: Fig. S2A, B). These findings suggest that MRE11A negatively regulates MMR activity, albeit not to an extent that significantly impacts the stability of microsatellites within approximately 30 genome duplications.

### MRE11A is recruited to chromatin by MMR proteins

**Fig. 4:**
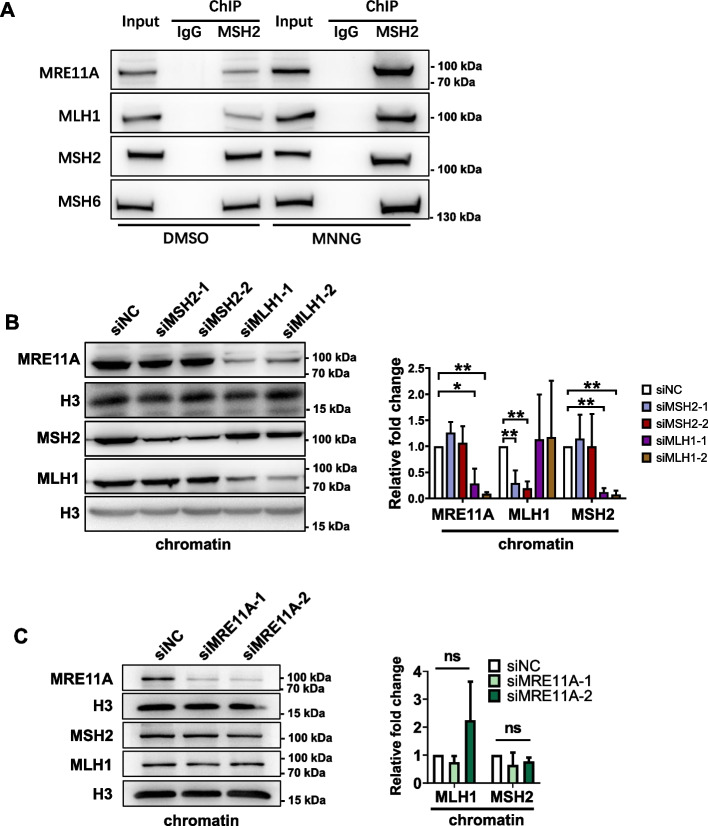
MRE11A is recruited to chromatin by MMR proteins. **A** Representative western blotting of chromatin proteins coprecipitated with MSH2 12 h after DMSO or 200 nM MNNG treatment. **B**, **C** Representative western blotting of the chromatin binding MSH2, MLH1, and MRE11A proteins after knockdown of MSH2, MLH1, and MRE11A independently. The right graphs show the quantification of the western blotting bands relative to siNC controls. Data shown as mean ± SD, *P < 0.05, **P < 0.01, ***P < 0.001, using unpaired two-tailed Student’s *t* test

Given the regulatory role of MRE11A in MMR activity, we next aimed to examine its potential interaction with the MMR machinery. To this end, we conducted chromatin immunoprecipitation assays using an MSH2 antibody. In the absence of MNNG treatment, we observed the expected recruitment of MSH6 with MSH2 on chromatin, while the coprecipitation levels of MLH1 and MRE11A were found to be relatively low (Fig. 4A). However, after 12-h exposure to MNNG, we detected co-recruitment of more MRE11A and MLH1 on chromatin with MSH2 than in absence of MNNG, suggesting an interaction between MRE11A and MLH1 (Fig. 4A). Furthermore, MLH1 deficiency, but not MSH2 deficiency, resulted in a significant decrease in chromatin-bound MRE11A (*P* < 0.001) (Fig. 4B). Additionally, we observed that MRE11A deficiency did not influence the levels of chromatin-bound MLH1 and MSH2 (Fig. 4C). These findings and those of previous studies collectively suggest that MRE11A participates in the MMR pathway through interaction with MLH1 [23, 24].

### MRE11A negatively regulates PMS2 protein levels

**Fig. 5:**
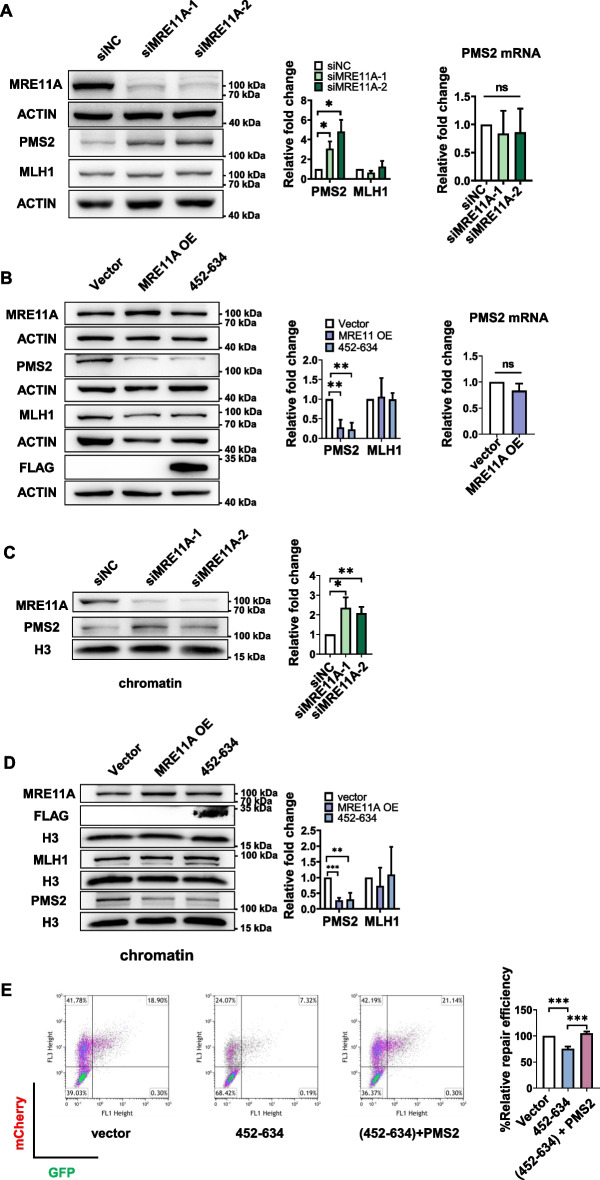
MRE11A negatively regulates PMS2 levels. Representative western blotting of the indicated protein levels in whole cells (**A**, **B**) or on chromatin (**C**, **D**) with MRE11A knockdown/overexpression or expression of flag-tagged 452–634AA of MRE11A. The right graphs show the quantification of the western blotting intensities relative to siNC or empty vector controls. The changes in the mRNA levels of PMS2 after MRE11A knockdown (**A**) or overexpression (**B**) were quantified using qPCR. Representative scatter plots of flow cytometry analysis of cells expressing GFP or mCherry, reflecting MMR repair efficiencies of cells expressing 452–634AA of MRE11A with/without PMS2 overexpression (**E**). The MMR repair efficiency was calculated as the ratio of the number of GFP-positive cells to mCherry-positive cells, and the quantification results relative to siNC or empty vector controls are shown in the right graphs. Data shown as mean ± SD, *P < 0.05, **P < 0.01, ***P < 0.001, using unpaired two-tailed Student’s *t* test

The interaction domain of MLH1 with MRE11A overlaps with its interaction domain with PMS2, which is located at the C-terminal region of MLH1 encompassing amino acids 495–756AA. Additionally, mutations in K618, K616, or L574 of MLH1 have been shown to disrupt its interactions with both MRE11A and PMS2 [23, 24, 39, 40]. This suggests the possibility that MRE11A may compete with PMS2 for binding to MLH1, thereby reducing the levels of MLH1–PMS2 complex on chromatin and subsequently interfering with MMR activity. To test this hypothesis, we assessed the protein levels of PMS2 and observed a decrease (*n* = 3; *P* = 0.0032) upon MRE11A overexpression and an increase (*n* = 3, 3; *P* = 0.0086, 0.0050) upon MRE11A depletion, while the mRNA levels remained unchanged (Fig. 5A, B). This can be attributed to the instability of PMS2 protein in the absence of MLH1 binding [41, 42]. Moreover, the levels of PMS2 on chromatin increased (*n* = 3, 3; *P* = 0.012, 0.0043) upon MRE11A depletion and decreased (*n* = 3; *P* < 0.001) upon MRE11A overexpression (Fig. 5C, D), while MLH1 levels remained unchanged (Fig. 4C and 5A, B, D). Notably, we found that overexpression of PMS2 restored the decreased MMR activity (*n* = 3; *P* = 0.0029) resulting from MRE11A overexpression, while overexpression of PMS2 alone did not significantly influence MMR activity (Fig. 3B, Additional file 1: Fig. S3), indicating that MRE11A expression interfered with MMR activity by downregulating PMS2 levels. Furthermore, we found that overexpression of MRE11A peptide 452–634AA, the binding motif with MLH1 [23, 24], can decrease PMS2 protein level in whole cell extracts (*n* = 3; *P* = 0.0013) as well as on chromatin (*P* = 0.0046) (Fig. 5B, D). Overexpression of the MRE11A peptide 452–634AA also downregulated MMR activity (*n* = 3; *P* < 0.001), which could be rescued by PMS2 overexpression (*n* = 3; *P* < 0.001) (Fig. 5E).

**Fig. 6:**
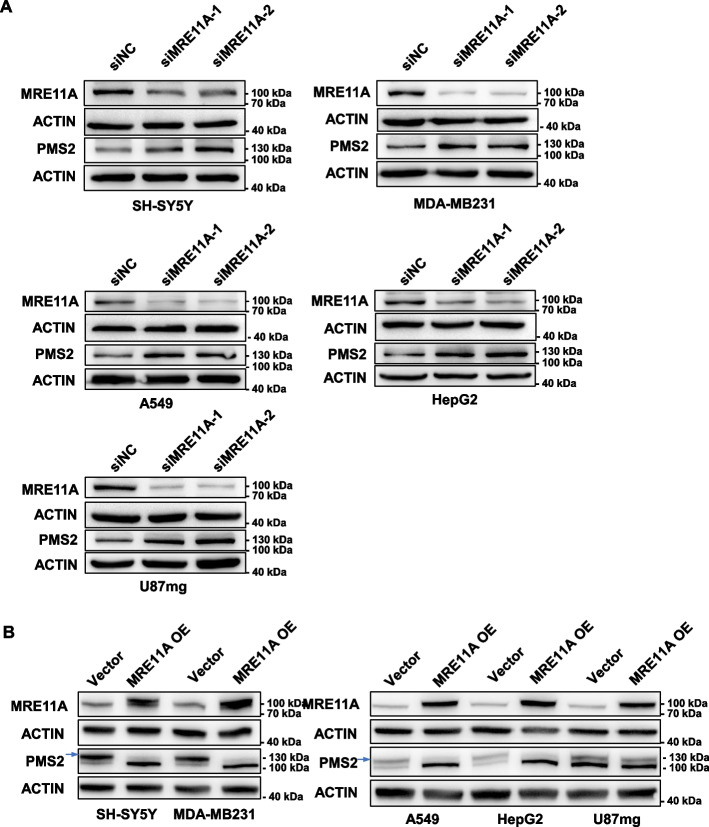
MRE11A levels negatively correlate with PMS2 levels in various cell lines. Representative western blotting of the PMS2 protein levels in whole cell lysates after MRE11A knockdown (**A**) or overexpression (**B**) in the indicated cell lines. The blue arrow indicates the PMS2 bands in the context of MRE11A overexpression

To assess whether the regulatory role of MRE11A on PMS2 levels extends to other cell lines, we depleted MRE11A in SH-SY5Y (neuroblastoma), MDA-MB231 (breast cancer), A549 (lung cancer), HepG2 (liver carcinoma), and U87mg (glioblastoma) cells. Consistently, the results demonstrated that MRE11A knockdown in these cell lines also led to an upregulation of PMS2 levels (Fig. 6A). Moreover, MRE11A overexpression in these cell lines, except for U87mg cells, led to dramatic downregulation of PMS2 levels (Fig. 6B). These findings provide additional evidence that the regulatory effect of MRE11A on PMS2 is not limited to HeLa cells and suggest a broader role of MRE11A in interfering with MMR activity by competing with PMS2 for binding to MLH1.

## Discussion

Mismatch repair is a highly conserved physiological process that plays an irreplaceable role in maintaining genomic integrity, spanning from prokaryotes to eukaryotes. In prokaryotes, multiple helicases and nucleases, such as Exo1, ExoVII, ExoX, and RecJ, contribute to the removal of mispaired bases in the newly synthesized strand. In contrast, exonuclease 1 (EXO1) has been identified as the sole exonuclease involved in eukaryotic MMR [43]. However, recent investigations have revealed that depletion of EXO1 in yeast or human cells only results in a modest decrease in MMR activity. This suggests the presence of alternative factors involved in the excision step of the process [16, 17, 44–46]. To date, studies have demonstrated that the strand displacement activity of DNA Polδ, WRN helicase, and FAN1, or the synergistic effect of multiple nucleases, may act as secondary factors to EXO1 in the MMR process [46–48]. Previously, we conducted high-throughput screening to identify novel MMR nucleases or helicases. We employed cellular sensitivity to low-dose MNNG, which is an SN1 alkylation reagent, as a readout to assess MMR activity [18]. Among the candidates, we observed that knockdown of MRE11A resulted in increased sensitivity of HeLa cells to MNNG, suggesting a potential negative regulation of MMR activity by MRE11A. This finding was unexpected, as a previous study by Her's group reported that MRE11A-deficient cells exhibited microsatellite instability on artificial substrates and displayed downregulated 3′ nick-directed MMR activity in vitro [24]. Considering that MRE11A possesses 3′–5′ exonuclease activity and that there are two reports indicating its interaction with MLH1, it is reasonable to hypothesize that MRE11A may serve as an alternative nuclease in the MMR pathway [23–25, 49]. However, two other reports showed that inactivation of MRE11A in eukaryotes did not affect MMR activity [30, 50]. Given the presence of conflicting results and the identification of MRE11A as a candidate in our screening assay, here we conducted a comprehensive investigation of MRE11A to validate its role in human MMR.

To validate the results obtained from the screening assay, we employed two different siRNAs to transiently downregulate the expression of MRE11A and subsequently assessed the sensitivity of cells to MNNG using both short-term and long-term clonogenic assays. Additionally, since MRE11A has been implicated in the repair of replication fork collapse in the second round of the cell cycle following MNNG treatment, we aimed to elucidate the specific role of MRE11A in MMR. To achieve this, we evaluated the extent of CHK1 phosphorylation and the number of 53BP1 foci within the first cell cycle after MNNG treatment. Furthermore, we conducted an MMR activity assay, which provided direct evidence of a negative correlation between MRE11A levels and MMR activity. Additionally, we demonstrated that MRE11A could be recruited to chromatin in association with MLH1 and that it interfered with the binding of PMS2 to MLH1. This interference led to a decrease in the levels of the MLH1–PMS2 complex on chromatin, ultimately resulting in the negative regulation of MMR activity by MRE11A.

MRE11A exhibits multifaceted functions in DNA repair and metabolism, encompassing the repair of double-strand breaks (DSBs) as well as the processing of stalled or collapsed replication forks [51–55]. While we observed increased DNA damage signals in MRE11A knockdown cells 12 h after MNNG treatment and strong recruitment of MRE11A to chromatin by MLH1, it is important to acknowledge the potential alternative roles of MRE11A in processing DNA lesions or managing replication stress. In fact, a previous report demonstrated that HeLa cells experienced replication stress and exhibited delayed S phase progression in the first cell cycle following MNNG treatment [37]. Therefore, it is possible that the observed increase in DNA damage signals could be attributed to inadequate processing of replication stress in the absence of MRE11A. However, the specific DNA damage response to replication stress following MNNG treatment is still not fully understood. It is worth noting that, thus far, the repair of ^O(6)Me^G/T by MMR remains the leading cause for the activation of DNA damage signals within the first cell cycle.

In our study, we observed that MRE11A has a negative regulatory effect on MMR activity, as evidenced by assays measuring repair mismatches on artificial substrates in cells. To further validate our findings, we conducted microsatellite instability (MSI) analysis, but we did not observe any instability in five clinically verified microsatellites. Microsatellites are repetitive sequences, typically consisting of one to six base pairs, found in the genome. Replication of microsatellite sequences is known to be inefficient for DNA polymerases, leading to the occurrence of replication slippages, which are then corrected by the MMR system to prevent the occurrence of insertion/deletion mutations. MMR-deficient cells have a higher probability of stochastically accumulating mutations in microsatellite sequences, resulting in high microsatellite instability (MSI). It has been established that the prevention of MSI requires nearly complete MMR activity, as inactivation of key MMR components such as MSH2 and MLH1 has been shown to result in high levels of MSI [56]. However, it should be noted that deletion of EXO1 does not necessarily cause MSI [16, 57, 58]. Therefore, while MSI has traditionally been considered the hallmark of MMR deficiency, it is possible that cells with decreased MMR activity may not develop MSI within a limited number of genome duplications. This could explain why we could not detect MSI in MRE11A-deficient cells. It is worth considering that sensitive detection methods, such as next-generation sequencing (NGS)-based MSI testing, may be needed to detect MSI in cells with decreased MMR activity.

We and other researchers have recently identified several factors, such as SLX4, CNOT6, HDAC6, and FAN1, that negatively regulate MMR activity [18, 59–61]. In addition to these findings, we present here that MRE11A inhibits MMR repair by competing with PMS2 for complex formation with MLH1. The identification of these negative MMR regulators suggests that cells employ a mechanism to prevent potential damage caused by imbalanced MMR activity. One well-known consequence of imbalanced MMR is the expansion of triplet nucleotide repeats, which has mutagenic effects and can lead to the onset of various human diseases, including Fragile X syndrome, Huntington’s disease, and spinocerebellar ataxia [62]. Excessive MMR activity is presumed to cause cellular hypersensitivity to alkylation damage and unnecessary processing of noncanonical DNA structures, resulting in chromatin instability. Indeed, studies have shown that overexpression of MSH3, MLH1, or PMS1 leads to downregulation of MMR activity or hypermutational phenotypes in eukaryotic cells [63–65]. Furthermore, clinical studies have indicated a correlation between the overexpression of MMR proteins and increased tumor aggressiveness or poor prognosis in various types of cancer [66–74]. Therefore, the negative regulators of MMR play a crucial role in maintaining a critical level of MMR to ensure genomic stability and metabolic homeostasis.

The presence of gaps or nicks on DNA strands containing mismatches is crucial for the correction of these mismatches. In the canonical MMR pathway, the endonuclease activity of PMS2 is activated upon the formation of the MLH1–PMS2 heterodimer, leading to the creation of nicks adjacent to the mismatches. These nicks serve as entry points for the exonuclease. Therefore, a decrease in PMS2 protein levels can severely compromise MMR activity [75]. In this study, we demonstrate that MRE11A competes with PMS2 for binding to MLH1, resulting in the interference of MMR activity by negatively regulating the levels of PMS2 or MLH1–PMS2 on chromatin. However, it is worth noting that, while the overexpression of MRE11A significantly decreases the level of chromatin-bound PMS2 protein, it only mildly, albeit statistically significantly, downregulates MMR activity. This observation suggests the possible backup role of MRE11A as an endonuclease, although not as efficient as PMS2, in the MMR pathway.

## Conclusions

**Fig. 7:**
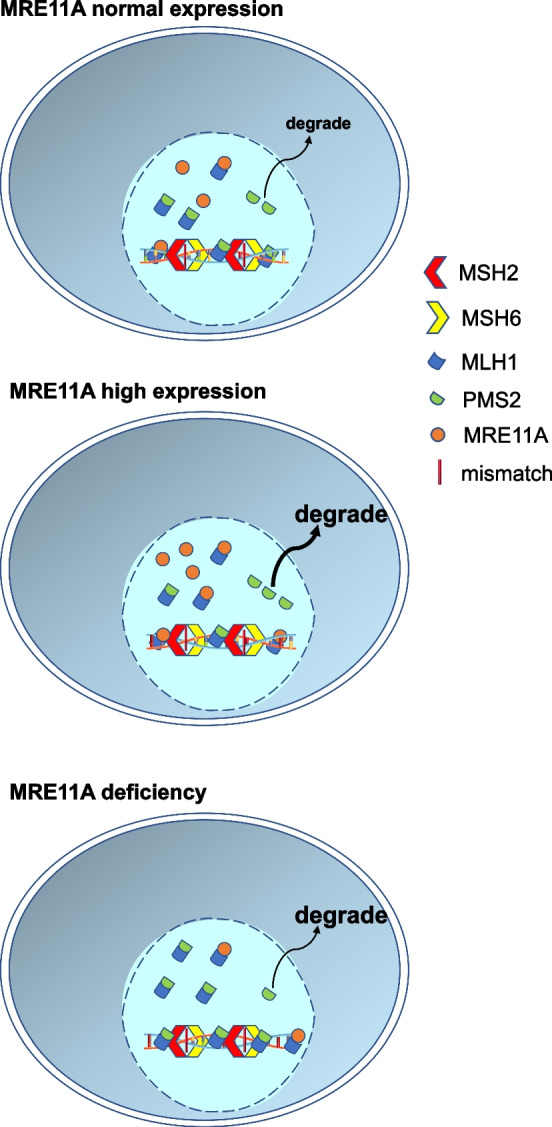
Schematic summary of the study. In naïve cells, a proportion of MRE11A may interact with MLH1 but does not interfere with the proper interaction between intrinsic PMS2 and MLH1. In MRE11A-overexpressing cells, excess MRE11A occupied the binding site of PMS2 to MLH1, leading to the degradation of unbound PMS2 and decreased MLH1·PMS2 heterodimer on chromatin, consequently compromising MMR activity. In MRE11A-deficient cells, more intrinsic PMS2 binds to MLH1, leading to increased MMR activity and thus increased sensitivity to MNNG treatment

Our study identified MRE11A as a novel negative regulator of MMR activity. Based on our findings and previous research, we propose that the binding of MRE11A to MLH1 obstructs the binding of PMS2 to MLH1, resulting in the downregulation of MLH1–PMS2 heterodimers on chromatin (Fig. 7). We speculate that the competition between PMS2 and MRE11A for MLH1 binding serves as a regulatory mechanism to maintain balanced MMR activity. Further investigations should aim to elucidate the biological significance of the interaction between MRE11A and MLH1, not only in regulating MMR activity but also in alternative DNA metabolism pathways, such as double-strand break repair and processing of replication stress, for the maintenance of genomic stability.

### Supplementary Information

**Additional file 1:** **Figure S1.** MRE11A overexpression decreases levels of DNA damage signals 12 h after MNNG treatment. **A** The representative western blotting of the phosphorylation levels of CHECK1 12 h after DMSO or 200 nM MNNG treatment. The alternation of phosphorylation level was calculated as the p-CHECK1 level to total CHECK1 protein after 200nM MNNG treatment minus that with only DMSO treatment. Right graph showed the quantification of proteins level changes relative to siNC. **B** Representative Immunofluorescent pictures of the 53BP1 foci in G1 phase 12 h after DMSO or 200 nM MNNG treatment. Right graph showed the quantification of the number of 53BP1 foci per cell in G1 phase (CYCLINA +). Data shown as mean ± SD, *n* = 3, *p < 0.05, **p < 0.01, ***p < 0.001, using unpaired two-tailed Student’s *t* test. **Figure S2.** MRE11A alternations does not induce microsatellite instability. **A** Capillary electrophoresis of PCR amplification products of indicated microsatellite gene loci was used for MSI test. Each group included ten samples, and 293T cell was set as positive control; only representative analysis results are shown. **B** Western blotting results of MLH1 levels in Hela cells and 293T cells. **Figure S3.** PMS2 overexpression does not influence MMR repair efficiency in HeLa cells. Left pictures represented the scatter plots of cells cotransfected with GFPheteroduplex and mCherry plasmids described in “Materials and Methods” section. The *x*-axis and *y*-axis represent the signal intensities of GFP and mCherry, respectively. The MMR repair efficiency was calculated as the ratio of the number of GFP-positive cells to mCherry-positive cells, and the quantification results relative to siNC or empty vector controls are shown in the right graphs. Here, cells were transfected with empty vector (Vector) or PMS2 expression vector (PMS2 OE) followed by GFP-heteroduplex and mCherry plasmids cotransfection after 2 days. Next day, the cells were subjected to flow cytometry for GFP and m-cherry signal analysis.

## Data Availability

The data that support the results of this study are available on request from the corresponding author (Dekang Liu) upon reasonable request.

## Abbreviations

ChIP: Chromatin immunoprecipitation
DSBs: DNA double-strand breaks
MSI: Microsatellite instability
MMR: DNA mismatch repair
MNNG: *N*-methyl-*N*′-nitro-*N*-nitrosoguanidine
RT-qPCR: Quantitative real-time polymerase chain reaction
