# Supplementary material for: MRE11A: a novel negative regulator of human DNA mismatch repair
Source: Cell Mol Biol Lett. 2024 Mar 14;29:37. doi: 10.1186/s11658-024-00547-z (PMC10938699; doi:10.1186/s11658-024-00547-z)
Supplement: Supplementary file 1 — Additional file 1: Figure S1. MRE11A overexpression decreases levels of DNA damage signals 12 h after MNNG treatment. A The representative western blotting of the phosphorylation levels of CHECK1 12 h after DMSO or 200 nM MNNG treatment. The alternation of phosphorylation level was calculated as the p-CHECK1 level to total CHECK1 protein after 200nM MNNG treatment minus that with only DMSO treatment. Right graph showed the quantification of proteins level changes relative to siNC. B Representative Immunofluorescent pictures of the 53BP1 foci in G1 phase 12 h after DMSO or 200 nM MNNG treatment. Right graph showed the quantification of the number of 53BP1 foci per cell in G1 phase (CYCLINA +). Data shown as mean ± SD, n = 3, *p < 0.05, **p < 0.01, ***p < 0.001, using unpaired two-tailed Student’s t test. Figure S2. MRE11A alternations does not induce microsatellite instability. A Capillary electrophoresis of PCR amplification products of indicated microsatellite gene loci was used for MSI test. Each group included ten samples, and 293T cell was set as positive control; only representative analysis results are shown. B Western blotting results of MLH1 levels in Hela cells and 293T cells. Figure S3. PMS2 overexpression does not influence MMR repair efficiency in HeLa cells. Left pictures represented the scatter plots of cells cotransfected with GFPheteroduplex and mCherry plasmids described in “Materials and Methods” section. The x-axis and y-axis represent the signal intensities of GFP and mCherry, respectively. The MMR repair efficiency was calculated as the ratio of the number of GFP-positive cells to mCherry-positive cells, and the quantification results relative to siNC or empty vector controls are shown in the right graphs. Here, cells were transfected with empty vector (Vector) or PMS2 expression vector (PMS2 OE) followed by GFP-heteroduplex and mCherry plasmids cotransfection after 2 days. Next day, the cells were subjected to flow cytometry for GFP and m-cherr [file 11658_2024_547_MOESM1_ESM.pdf]

- **Supplementary figure legends:**

- **Figure S1. MRE11A overexpression decreases levels of DNA damage signals 12h after MNNG treatment. (A)** The representative western blotting of the phosphorylation levels of CHECK1 12 hours after DMSO or 200nM MNNG treatment. The alternation of phosphorylation level was calculated as the p-CHECK1 level to total CHECK1 protein after 200nM MNNG treatment minus that with only DMSO treatment. Right graph showed the quantification of proteins level changes relative to siNC. **(B)** Representative Immunofluorescent pictures of the 53BP1 foci in G1 phase 12 hours after DMSO or 200nM MNNG treatment. Right graph showed the quantification of the number of 53BP1 foci per cell in G1 phase (CYCLINA+). Data are shown as mean ± SD, $n = 3$, * $p < 0.05$, ** $p < 0.01$, *** $p < 0.001$, using unpaired two-tailed Student's t test.

- **Figure S2. MRE11A alternations does not induce microsatellite instability. (A)** Capillary electrophoresis of PCR amplification products of indicated microsatellite gene loci was used for MSI test. Each group included ten samples and 293T cell was set as positive control and only representative analysis results were shown. **(B)** Western blotting results of MLH1 levels in Hela cells and 293T cells.

- **Figure S3. PMS2 overexpression does not influence MMR repair efficiency in HeLa cells.** Left pictures represented the scatter plots of cells co-transfected with GFP-heteroduplex and mcherry plasmids described in materials and methods. The x-axis and y-axis represented the signal intensities of GFP and mcherry respectively. The MMR repair efficiency was calculated as the ratio of the number of GFP positive cells to mcherry positive cells, and the quantification results relative to siNC or empty vector controls were in the right graphs. Here, cells were transfected with empty vector (Vector) or PMS2 expression vector (PMS2 OE) followed by GFP-heteroduplex and mcherry plasmids co-transfection after two days. Next day, the cells were subjected to flow cytometry for GFP and m-cherry signals analysis.

Figure S1

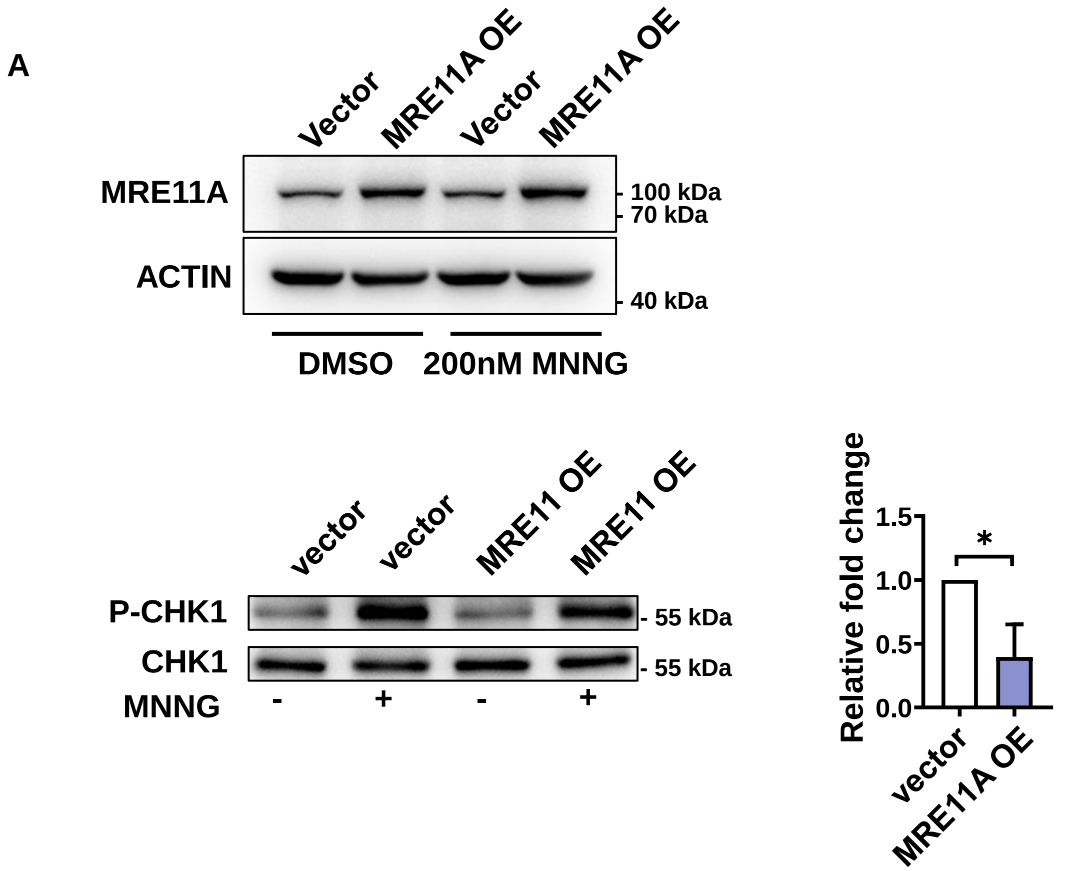
A
Vector
MRE11A OE
Vector
MRE11A OE
MRE11A
ACTIN
100 kDa
70 kDa
40 kDa
DMSO
200nM MNNG
vector
vector
MRE11 OE
MRE11 OE
P-CHK1
CHK1
MNNG
-
+
-
+
55 kDa
55 kDa
Relative fold change
1.5
1.0
0.5
0.0
*
vector
MRE11A OE

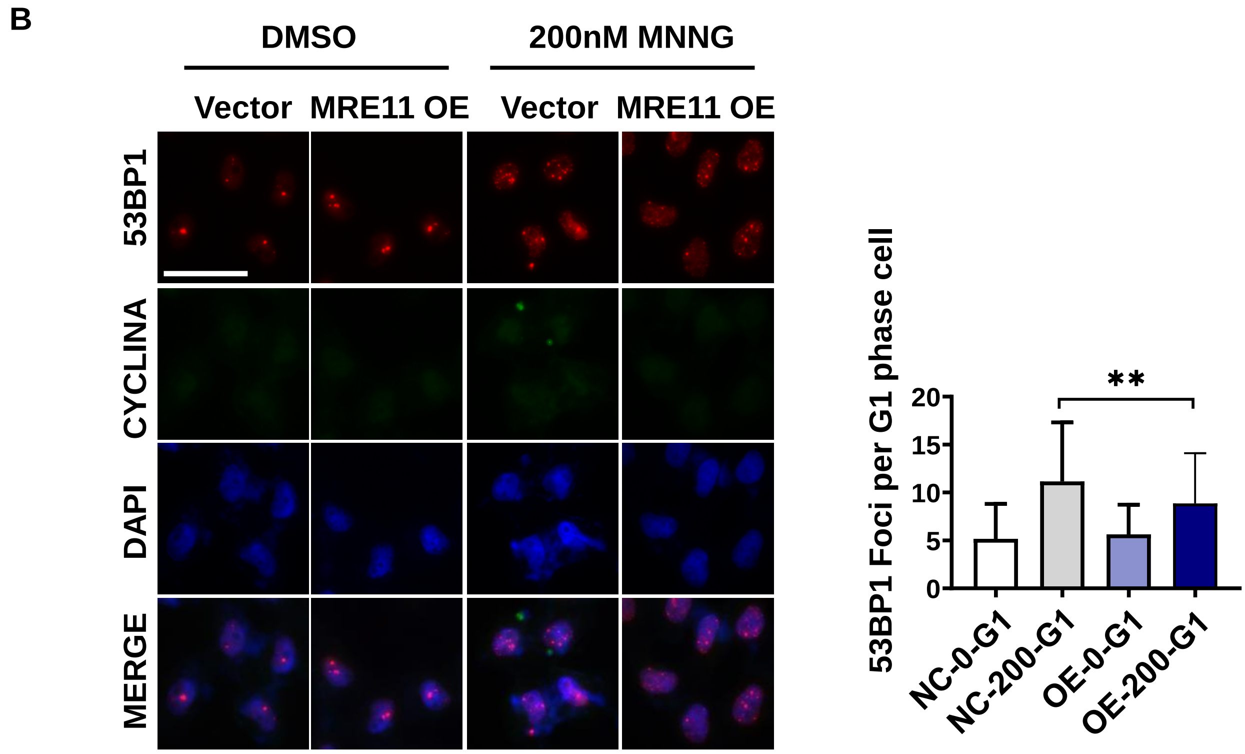
B
DMSO
200nM MNNG
Vector
MRE11 OE
Vector
MRE11 OE
53BP1
CYCLINA
DAPI
MERGE
53BP1 Foci per G1 phase cell
20
15
10
5
0
**
NC-0-G1
NC-200-G1
OE-0-G1
OE-200-G1

Figure S2

A

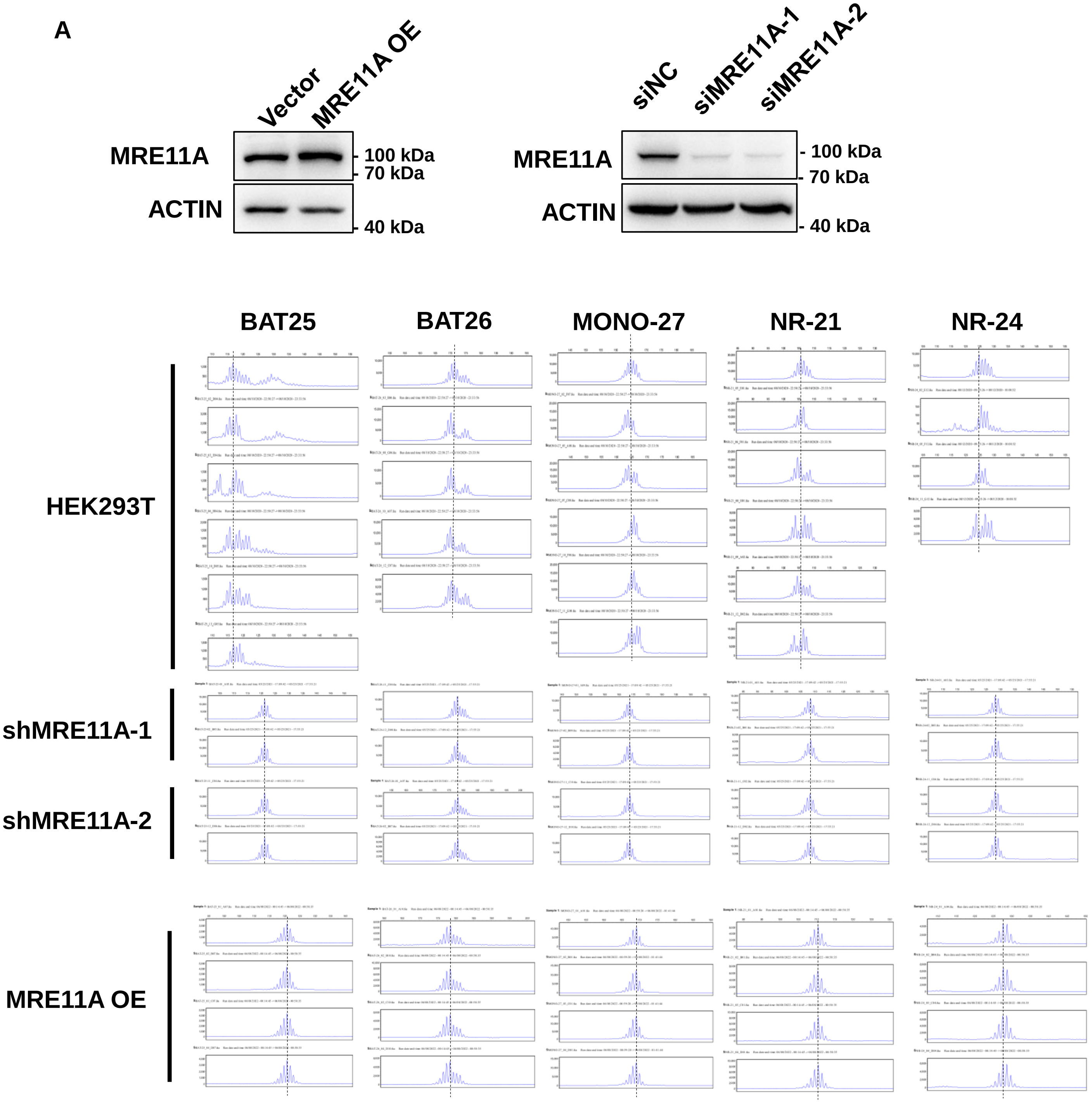

Vector
MRE11A OE
MRE11A
ACTIN
100 kDa
70 kDa
40 kDa
siNC
siMRE11A-1
siMRE11A-2
MRE11A
ACTIN
100 kDa
70 kDa
40 kDa
BAT25
BAT26
MONO-27
NR-21
NR-24
HEK293T
shMRE11A-1
shMRE11A-2
MRE11A OE

B

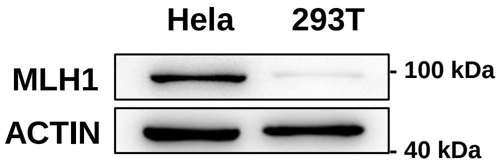

Hela
293T
MLH1
ACTIN
100 kDa
40 kDa

Figure S3

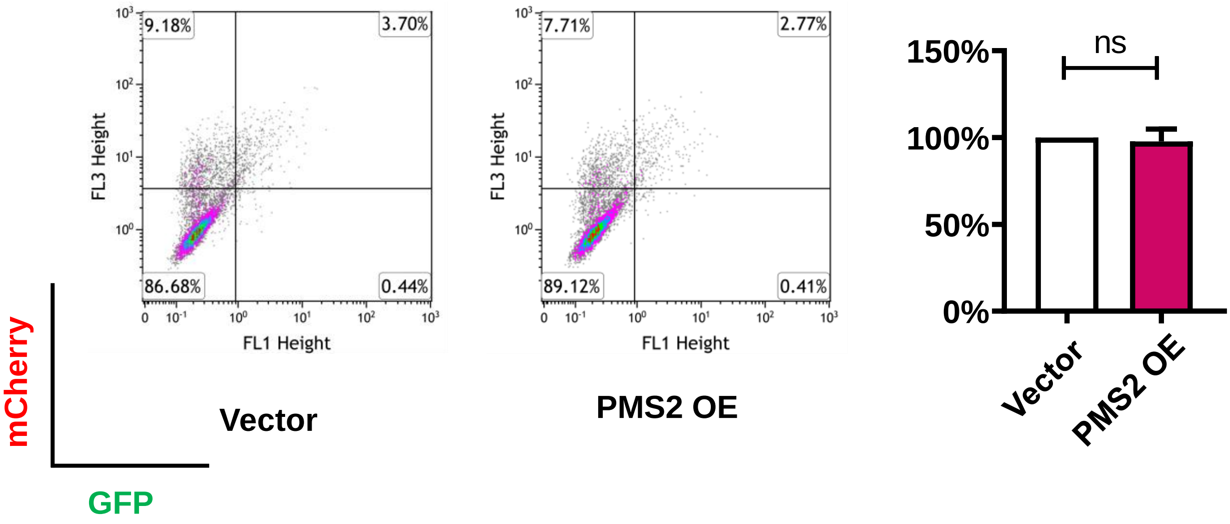
9.18%
3.70%
86.68%
0.44%
FL3 Height
FL1 Height
Vector
7.71%
2.77%
89.12%
0.41%
FL3 Height
FL1 Height
PMS2 OE
mCherry
GFP
ns
150%
100%
50%
0%
Vector
PMS2 OE
